# Non-monetary narratives motivate businesses to engage with climate change

**DOI:** 10.1007/s11625-023-01386-1

**Published:** 2023-07-10

**Authors:** Christopher Luederitz, Animesh Animesh, Katrin Rohrbacher, Tiange Li, Andrew Piper, Catherine Potvin, Dror Etzion

**Affiliations:** 1 Desautels Faculty of Management, McGill University, 1001 Sherbrooke St W, Montreal, QC H3A 1G5 Canada; 2 Faculty of Science, McGill University, Montreal, QC Canada; 3 Faculty of Arts, McGill University, Montreal, QC Canada; 4 Grossman School of Business, University of Vermont, Burlington, USA

**Keywords:** Sustainability communication, Narrative motivations, Storytelling, Climate action, Business, Small- and medium-sized enterprises (SMEs)

## Abstract

The dominant narrative to motivate business actors to take climate actions emphasizes opportunities to increase monetary gains, linking sustainability to the financial goals of these organizations. The prevalence of monetary motivations in sustainability communication among businesses, consultancies, academics and international organizations has made this narrative a truism in the private sector. We conducted an online, real-world, large-*n* experiment to evaluate the comparative effectiveness of different motivations using narrative communication. We show that non-monetary narratives highlighting prosocial or achievement motivations are 55% more effective in creating responses from businesses than narratives emphasizing monetary gains. These findings are robust across most narrative and audience characteristics, including age and language. Our findings suggest that communication towards business leaders around sustainability can be multi-pronged and should incorporate prosocial and achievement motivations aside from articulating potential financial benefits.

## Introduction

Global efforts to address the climate crisis require businesses, alongside governments, individuals, non-profits and other organizations, to act decisively to dramatically reduce emissions (Sachs et al. 2019). To motivate businesses to take action, climate change communicators have emphasized the need for narratives to accommodate and respond to the unique characteristics of this type of social actor (Bushell et al. 2017; Westman et al. 2019). Since businesses pursue monetary gain as a primary goal, it is generally taken for granted that the best way to incentivize these organizations towards social and environmental goals is by articulating a “business case” for why such action is desirable (King and Pucker 2021).

A widely used narrative for companies to pursue sustainability argues that building a business case for sustainability would lead to success in the form of financial profitability or organizational longevity. Communications that emphasize the business case for sustainability are honed and disseminated by consultancies (e.g., McKinsey and Company 2011), academics (e.g., Kramer and Porter 2011) and international organizations (e.g., World Bank 2014) so much so that they have become almost axiomatic (Riedy 2022). Proponents of this narrative point to business strategies around differentiation, risk management, product development and business model innovation that contribute to desirable social and environmental outcomes while improving profitability (Nidumolu et al. 2009; Vishwanathan et al. 2020).

Past research has called into question the effectiveness of communication focused exclusively on the business case to engage companies in climate action. Several laboratory experiments have revealed that managers are skeptical of claims about sustainability action yielding desirable financial results (Hafenbradl and Waeger 2017). Research has also suggested that business case language can “backfire,” leading managers to judge investment decisions less favourably (Rode et al. 2021). Considering these findings, researchers have started studying alternative ways of communication, demonstrating, for example, how moral language can be more effective than monetary language for encouraging businesses to undertake sustainability efforts (Mayer et al. 2019). Others have shown that awareness about climate change is linked to biospheric values (e.g., sentiments about protecting nature) and thus communication needs to appeal to such values to motivate climate action (Bouman et al. 2020). These findings suggest a need to move away from studying purely economic narratives and identify other effective narratives for business audiences.

A key challenge in studies trying to examine communication effectiveness is methodological. Research on responses to climate change messaging is typically restricted to novices in laboratory settings (e.g., Hafenbradl and Waeger 2017; Rode et al. 2021), whereas real-world studies are often limited to examining motivations in ongoing initiatives through observational studies (Williams et al. 2017). These circumstances have made it difficult to identify what motivations would be most effective to engage businesses for the first time in climate change efforts in real-world settings.

In this study, we examine the comparative effectiveness of different narratives for engaging businesses in real-world settings. For this purpose, we designed a real-world, large-*n* experiment on the social media platform Facebook and developed three distinct narratives to test engagement—namely, monetary, prosocial, or achievement motivations. Our research design featured real business owners as protagonists and made use of their own words for explaining why they take climate action. Our protagonists varied in terms of gender and age and we utilized Facebook demographic data to stratify our experiment by age and language. As such, our design allowed us to control for both protagonist and audience data.

Our research makes three significant contributions to the literature on sustainability communication. First, our findings shed light on the effectiveness of different forms of messaging for initially engaging businesses in climate change conversation. We show that interest in such communication is more strongly triggered by narratives that describe non-monetary motivations, such as protecting the environment or personal fulfillment. Second, our experimental design disentangles narratives that are often jumbled together in stories told by protagonists in real-world situations. We achieved this by randomly presenting our audiences with only one of three distinct, mutually exclusive narratives that we developed for each protagonist. Third, based on our findings, we offer practical guidance for sustainability communication, highlighting opportunities to employ multi-pronged messaging when communicating with businesses about climate action.

Below, we elaborate on the theoretical background of our study and outline how we developed our research design around the needs of small- and medium businesses, building on past research on narrative communication. In the “Methods”, we describe the experimental design and treatments before presenting our findings in “Results”. The discussion and conclusion draw attention to the key insights of our research and set forth practical guidelines for improving sustainability communication.

### Narrative communication and business sustainability

The use of narratives is a communications approach to promoting change, grounded in the arts and the humanities. (Moser 2016; De Meyer et al. 2021; Koch et al. 2021). As retellings of past actions, narratives are overarching accounts of events, providing a rationale for a course of action (Todorov 1981, pp. 41–51), facilitating causal reasoning on the part of readers and helping answer “why” questions when it comes to events in the world (Dahlstrom 2010; Graesser et al. 2021). Narrative communication has been demonstrated as a powerful form of persuasion for attaining desirable outcomes across different domains due to its focus on personalized accounts of events (Green 2006; Dahlstrom 2014; Sabherwal and Shreedhar 2022). Central to narratives is the explicit focus on protagonists to encode actions and motivations (Dahlstrom 2010; Markowitz and Guckian 2018), garnering interest from audiences and enabling them to develop strong affective attachments to the story (Green and Brock 2000; Morris et al. 2019). This positive affect can, in turn, help audiences support and enact the assumptions and arguments embedded in the narratives (Downs 2014; Jones 2014).

Research on narratives in sustainability contexts has revealed the power of stories to inspire climate actions (Jones and Crow 2017; Whitmarsh and Corner 2017; Sangalang and Bloomfield 2018; Veland et al. 2018; Gustafson et al. 2020). Narrative communication has been used across a broad array of subject areas, including but not limited to climate change (Moezzi et al. 2017; Whitmarsh and Corner 2017), urban resilience (Borie et al. 2019), environmental governance (Denton 2017), and food production (Cusworth et al. 2021; Paprocki 2022). Particularly persuasive narratives appear to emerge in a decentralized manner and to feature relatable protagonists (Bevan et al. 2020). In particular, authentic human stories revolving around real events and actions are captivating to audiences if tailored messaging is employed (Markowitz and Guckian 2018; Morris et al. 2019; Gustafson et al. 2020).

Narratives differ from other forms of sustainability communication that have focused on explaining environmentally harmful activities by imparting facts, with the expectation that providing such information leads individuals to modify their behavior (Fischhoff 2013). It is now clear that such communication, founded upon the information deficit model, is limited in its ability to inspire action (Boykoff 2019). Other forms of communication function by short-circuiting the information deficit problem through the usage of brief, acontextual messages such as signage, factual labels and prompts, with the aim of nudging people to enact desirable choices (Mertens et al. 2022). While nudging has proven advantageous in various contexts (Hummel and Maedche 2019), its effectiveness is limited for encouraging substantive actions (Hagmann et al. 2019). Although stories and narratives require more attention to process and internalization, their ability to spark change is potentially greater. Our study focuses on the use of climate narratives that target owners and managers of small businesses.

### Motivations for climate actions among small- and medium-sized enterprises

Clarifying the narratives that motivate small- and medium-sized enterprises (SMEs) to respond to climate change messaging is important because they constitute the bedrock of economies worldwide. For example, they make up 99% of all businesses in the European Union and contribute 60–70% of industrial pollution, while in Canada the more than 1.2 million SMEs emit 200 tons of carbon annually, which is equal to the emissions of the country’s transportation sector (Miller et al. 2011; Schmelzer 2015; Climate Smart 2018; ISED 2020). Despite their significant carbon footprint, it remains unclear how climate narratives can be used to create interest among SMEs to learn more about opportunities to take climate action.

From a practical point of view, SMEs are often financially fragile (Bartik et al. 2020) and improving sustainability performance could be a feasible avenue to support their bottom line (Malesios et al. 2018). Yet, these organizations lack the elaborate governance mechanisms and structured decision-making criteria that are common in large companies to assess and enact climate action (Stubblefield Loucks et al. 2010), and thus may be unaware of opportunities and benefits of doing so. At the same time, owners and founders of SMEs have a great degree of latitude in pursuing strategies of their choosing and, therefore, more opportunities to integrate personal values with business objectives (Williams and Schaefer 2013). Indeed, SME owners may be motivated to pursuing challenges and achieving goals related to the sustainability performance of their business (Schaefer et al. 2020), even without a clear economic or regulatory rationale for doing so. Research has also shown that while some SMEs pursue climate action because of financial opportunities, others readily respond to prosocial concerns because the owner cares for the well-being of their local community or natural environment (Kaesehage et al. 2019).

Our research examines the effectiveness of different motivations to generate interest from businesses in climate action. In particular, the comparative efficacy of monetary, achievement and prosocial values has not been assessed. Further, there is increasing consensus that examining different motivations requires their embedding in real event storytelling (Mayer et al. 2019). We advance this line of research by employing a major social media platform to conduct a real-world experiment for testing different narratives about business-led climate action centered around real people, their challenges and successes. Addressing this research gap offers not only theoretical advancements in the role of different values in communicating climate action, but also offers practical insights for communicators designing sustainability campaigns. Collectively, our study advances research on sustainability communication by providing evidence for the effectiveness of different motivations in engaging businesses in climate action (Koch et al. 2021).

## Methods

We ran our experiment as part of a multi-faceted action research collaboration between university researchers and creatives of a national media organization. The goal of the collaboration was to “Amplify in real time the actions of small businesses to accelerate tipping points that reveal opportunities in the new climate economy.” As part of our experiment, we created a dedicated project website, www.gopivot.org,^1^ to explore how SMEs respond to communication about climate action. To promote this project, we also created a short documentary, animations, and social media narratives on LinkedIn, Twitter, Instagram and Facebook. This action research setting enabled and informed our experimental design, as described below.

### Narrative creation

Prior research has identified three distinct motivations as to why a protagonist (i.e., business owner) might pursue climate action (Stern et al. 1993): monetary, prosocial and achievement (with the latter two jointly comprising non-monetary motivations):

1. *Monetary*: The protagonist is motivated by financial interests, such as making money, growing their business or being commercially successful. This is the standard “business case for sustainability” motivation.
2. *Prosocial*: The protagonist is motivated by altruistic and/or biosphere values (De Groot and Steg 2007), such as a concern for the environment, their children or their community.
3. *Achievement*: The protagonist is motivated by internal needs or desires (i.e., self-enhancing values) such as a drive to excel, love of a challenge or sense of duty. These forms of motivation are associated with entrepreneurial activity (Collins et al. 2004).

Our research design made use of narratives corresponding to each of these motivations to examine their effectiveness in eliciting interest from SMEs in climate change communication. The narratives we developed were based on real events and actors. We developed them after conducting semi-structured interviews with 13 Canadian SME owners (i.e., the protagonists of our narratives) to identify monetary, prosocial and achievement motivations in relation to why they realized climate action. The business owners are real individuals, each of whom has their own mix of reasons and motivations for engaging in climate action. Of the 13 business owner protagonists, seven were identified as male, six as female, two as visible minorities and two as Indigenous. These protagonists represented the agriculture, building, manufacturing and retail sectors, and seven of the ten provinces in Canada.

In a second step, based on the in-depth interviews, we developed narratives for each protagonist. The data that we collected through the interviews on each protagonist typically integrated more than one motivation. Therefore, we worked with professional copy editors to create three separate texts for each protagonist, one each for monetary, prosocial and achievement motivations. Thus, in total, we developed 39 article-based narratives (13 stories for each motivation), averaging 795 words per story. These stories were made accessible at the time of the experiment on the project’s website www.gopivot.org. All material included for this research can be found online in the Open Science Framework repository (https://osf.io/suyq5/) including the three different narratives for each protagonist.

### Experimental setup

**Fig. 1:**
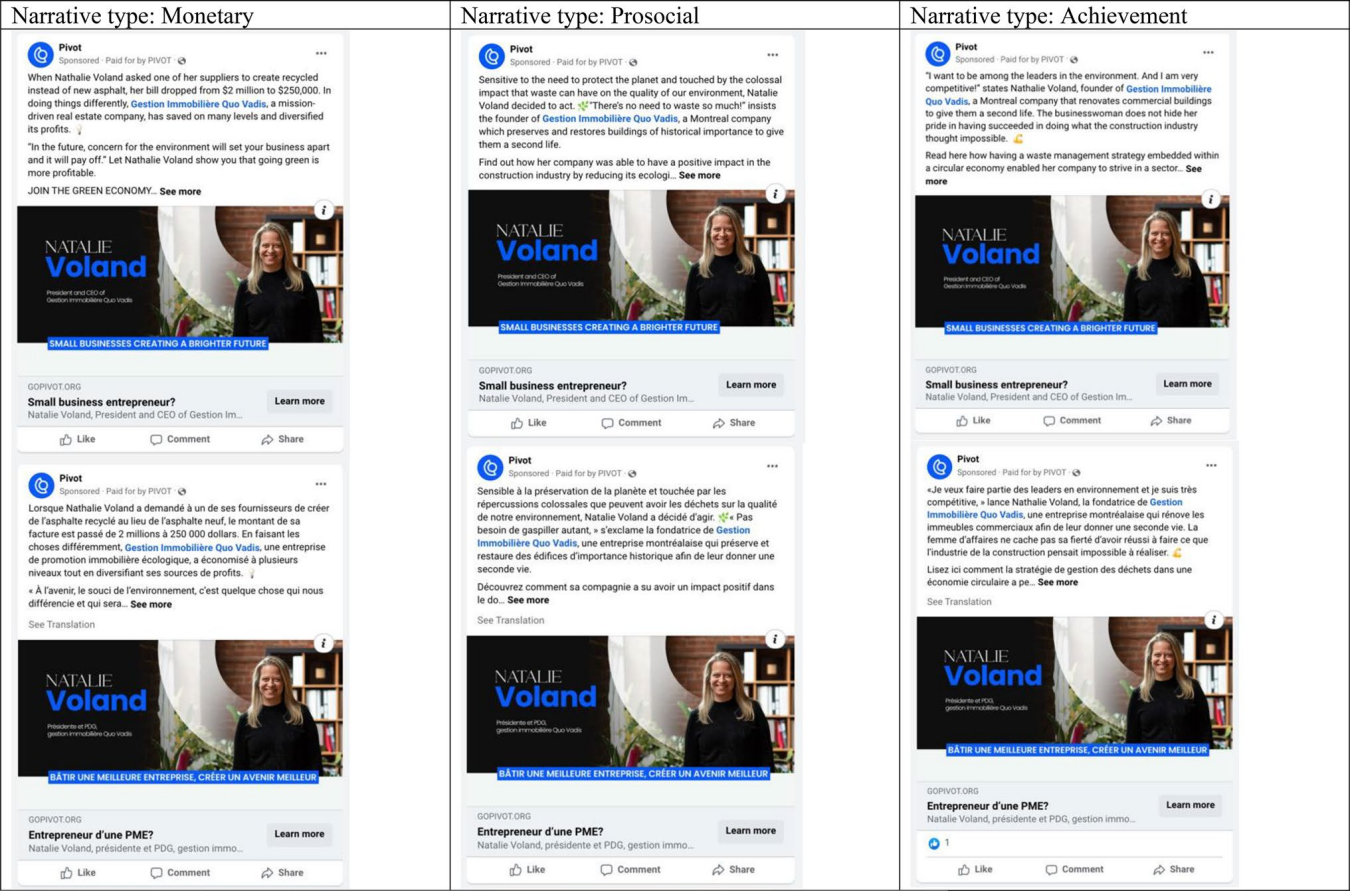
Display of the three narrative summaries for one specific protagonist on Facebook in English and French

We launched an experiment on Facebook to test the effectiveness of the three narrative motivations. Since it was not possible to portray the full version of the article-based narratives as part of the experimental setup on Facebook, we developed short summaries that conveyed the main motivation (we refer to these shorter versions as ‘narrative summary’ hereafter). Each narrative summary served as a “teaser” on Facebook, consisting of a concise description of one of the 39 narratives, averaging 76 words (see Fig. 1). Each narrative summary included five parts: (1) an explanation of the motivation why a business owner (the protagonist) acted on climate change, (2) a call for action targeting the audience to learn more about this narrative, (3) a reinforcement statement, combining the motivation and the call for action, (4) a picture of the protagonist and (5) a “learn more” button that referred people to the article-based narrative about the protagonist on the project’s website. All article-based narratives and summaries were written in both French and English. The research design for creating the narratives was authorized by the university’s Research Ethics Board Office.

#### Participant recruitment

The experimental setup and the Facebook infrastructure enabled us to distribute each narrative summary to a predefined target population. Given that Facebook does not require its members to accurately identify their jobs or business affiliations, we leveraged interest-based targeting offered by the Facebook platform to best reach individuals who are SME owners. Specifically, we targeted individuals who indicated to Facebook an interest in one or more of the following categories: small and medium enterprises, shop local, sustainability, business development, human resources, social innovation, risk management, entrepreneurship. We further limited our sample population to people residing in Canada to correspond with the context of the protagonist featured in the narratives. According to Facebook, the potential reach of this sample population was 800–900 k members. With this targeting strategy, we attempted to find a balance between over- and undersampling SME owners. Undersampling might occur if targeted audience members are SME owners, but do not indicate interest in the “Small and medium enterprises” category on Facebook. By contrast, the selection of a large number of interest-based categories would have created the risk of introducing Facebook members into our sample who are not SME owners. Our targeting criteria, therefore, attempted to balance out these two undesirable/extreme scenarios. It is important to note that while Facebook uses interest topics for targeting, it does not share this information, barring us from identifying, post hoc, the interest-based keywords of Facebook members to whom the narrative types were shown.

#### Data collection

The experiment was conducted over ten days (April 29–May 8, 2021) on Facebook to randomly show the narrative summaries to the target audience. The narrative summaries were presented in a true-to-life social media environment as individuals looked through their own personal Facebook Newsfeed. We employed the Ads Manager tool provided through the Facebook Business Suite application to set up our sample and conduct our experiment (Jilke et al. 2019).

We stratified our experiment into 5 blocks to target different age groups corresponding to Facebook’s 5 age categories (25–34, 35–44, 45–54, 55–56, and 65+). In each of these blocks, we placed 39 narrative summaries in English (13 protagonists* 3 narratives). We then replicated this experimental design and replaced the English narratives with the French translation to target English and French speakers separately. We used Facebook’s proprietary “Optimization for Ad Delivery” algorithm and selected the option of maximizing the target audience, in line with the outreach objectives of the action research collaboration. As a result, the 78 narratives (13 protagonists* 3 narrative types* 2 languages) may differ in terms of impressions (i.e., the number of audience members who were shown an ad). We have no reason to believe that this design choice created any systematic bias for/against any narrative type such that summaries of a given narrative were shown more/less frequently to individuals who were more likely to engage vis-a-vis another narrative. Moreover, in a post hoc Kolmogorov–Smirnov (K–S) test, we were able to confirm no biases in age between the samples that viewed the different narratives.

Facebook recorded whether audience members clicked on the various parts of the ads. We used the number of clicks as a proxy for the number of people interested in the narratives. We employed two variables to measure engagement: clicks on the hyperlinks (hereafter ‘link clicks’) and clicks on other parts of the narrative summary (hereafter ‘narrative clicks’) (Lee et al. 2018). Our primary measure, link clicks, was the number of clicks on hyperlinks that were part of the summary (i.e., the “learn more” button in Fig. 1, which led to the article-based narrative on the project's website). We supplemented this variable with narrative clicks, which measured all other clicks on the narrative (excluding link clicks). This encompassed clicks on (1) the “see more” button to read the entire narrative summary text, (2) the picture that was part of the narrative to enlarge it or (3) the PIVOT logo in the summary to visit the project’s Facebook profile. Our response rate of 0.57% for link clicks is higher than similar research employing Facebook to study, for example, political theories with 0.45% (Jilke et al. 2019) or health messaging with 0.24% (Reuter et al. 2021).

Separately, to determine the perceived age of each protagonist, we gave a group of eight students in sustainability programs the randomly assorted narrative summaries. Based on the ad picture, their task was to assign each protagonist to one of three categories (young: range 23–44 years old, middle-aged: range 45–65 years old, senior, range 65+). The estimation of the protagonists’ age was coded as “Protagonist age.”

#### Data analysis

To assess whether businesses were interested in the climate change narratives distributed through our experiment, we analyzed the effect of each narrative type and demographic characteristics—such as language and age—on audience engagement. Furthermore, we analyzed the perceived age and gender of the business owner protagonists that were featured in each narrative to assess their influence on audience engagement. For a detailed results report, see the online material in the Open Science Framework repository (https://osf.io/suyq5/).

To investigate the effect of each narrative type on the audiences’ likelihood to click on a narrative hyperlink (i.e., link clicks), we used a generalized linear mixed effects model. Exposure to a narrative summary is tracked by Facebook through impressions which refers to the number of individuals in the target audience in whose social media feeds the narratives appeared. The dependent variable for our analysis was either 0 (for no click) or 1 (for click) for each instance in which an audience member was exposed to one of the narrative summaries. To obtain this measure, we disaggregated the count data we received from Facebook into separate rows, with each row corresponding to an impression. We specified a logistic regression model with a random effects term to account for differences between individual protagonists. The mixed effects specification allowed us to consider each of the 13 protagonists as a distinct experiment in which we tested the effects of the three different motivations. Whether the $j$th impression belonging to protagonist $prot$ leads to an audience member reaction (i.e., link click) follows a Bernoulli distribution $B\left({\pi }_{\mathrm{prot},j}\right)$. In our model, the logit of the parameter ${\pi }_{prot,j}$ is assumed to depend on the narrative type that each summary incorporated, represented by a categorical variable, as well as a set of control variables. Formally, our model is:

$$\mathrm{logit}\left({\pi }_{\mathrm{prot},j}\right)= \mu + {U}_{\mathrm{prot}}+\sum_{k=\mathrm{0,1}}{\beta }_{k}{X}_{\mathrm{prot},j}^{k}+\sum_{l=\mathrm{0,1}}{\gamma }_{l}{Y}_{\mathrm{prot}}^{l}+\sum_{m=\mathrm{0,1}}{\delta }_{m}{Z}_{\mathrm{prot},j}^{m}+{E}_{\mathrm{prot},j}.$$

Here, prot = 0,…,12 corresponds to each of the 13 protagonists. X is our main independent dummy variable indicating narrative types (i.e., monetary, prosocial, achievement motivations). *Y* is a vector of control variables pertaining to the protagonists, capturing protagonists’ age and gender. *Z* is a vector of control variables pertaining to the Facebook audience, capturing audience age and language as per the 10 Facebook blocks described above. ${U}_{\mathrm{prot}}$ is the random intercept specific to each protagonist, and *E* is an error term capturing all remaining variance. We ran our analysis using the lme4 package in R.

As a robustness test, we modelled the effect of narrative type on our second dependent variable, namely narrative clicks (which excludes link clicks). This variable was collected in the same manner as the other data used for our main analysis. Because each member of the target audience could click more than once on a given narrative, we deemed a logit model inappropriate. We thus fit a generalized mixed effects linear model with a negative binomial link function to model this dependence, controlling for the same set of covariates as in the model described above. Instead of analyzing the odds ratio as done for the logistic regression model, we measured the incident rate ratio to examine multiple, non-mutually exclusive interactions within in one narrative. We chose a binomial distribution to account for overdispersion (rather than its special case, a Poisson distribution, as is often done for count data). Raw data and materials and the custom codes for analysis are available in the Open Science Framework repository.

## Results

Collectively, the 39 narrative summaries, which were shown to 5 different age groups in English and French on Facebook, gained 94,712 impressions. In total, we recorded 540 clicks on hyperlinks (= “link clicks”) and 1773 clicks on other items (= “narrative clicks”) across the three narrative types.

### Non-monetary narratives are more effective

Results of our logit model are presented in Table 1. We first compared responses to the monetary narrative with the two non-monetary narratives (i.e., prosocial and achievement narratives). We find that when communicating climate stories to business audiences, the response rate is higher if non-monetary motivations of protagonists are articulated. Specifically, narrative summaries featuring protagonists’ concern for the environment or their passion for addressing challenges related to climate change generated better engagement than monetary narratives. Taken together, non-monetary narratives were 54.8% more effective in garnering interest from business audiences in matters of climate change [odds ratio (OR) = 1.54, *p* = 0.015, 95% confidence interval (CI) = (1.09, 2.17)].

**Table 1:** Logistic mixed-effects models predicting audience engagement as measured by link clicks (*n* = 94,712)

Predictors	Model 1		Model 2		Model 3		Model 4	
	OR (SE)	95% CI	OR (SE)	95% CI	OR (SE)	95% CI	OR (SE)	95% CI
Protagonist age	0.83 (0.13)	0.61–1.11	0.84 (0.09)	0.68–1.04	0.83* (0.06)	0.71–0.96	0.82* (0.07)	0.70–0.96
Protagonist gender	0.96 (0.19)	0.65–1.42	0.98 (0.14)	0.74–1.31	1.07 (0.12)	0.86–1.33	1.07 (0.12)	0.86–1.32
Language	1.00 (0.11)	0.81–1.23	1.04 (0.11)	0.85–1.28	1.06 (0.10)	0.89–1.28	1.06 (0.10)	0.88–1.28
Audience age			1.38*** (0.05)	1.29–1.48	1.38*** (0.05)	1.29–1.47	1.38*** (0.05)	1.29–1.47
Non-monetary narratives					1.54* (0.27)	1.09–2.17		
Prosocial narrative							1.56* (0.29)	1.09–2.24
Achievement narrative							1.51* (0.28)	1.05–2.18

*OR* odds ratio, *SE* standard error, *CI* confidence interval

*p < 0.05, **p < 0.01, ***p < 0.001. ‘Non-monetary narratives’ is the ratio of the likelihood of clicking on achievement narrative or prosocial narrative against the likelihood of clicking the monetary narrative. ‘Prosocial narrative’ is the ratio of the likelihood of clicking on prosocial narrative against the likelihood of clicking the monetary narrative. ‘Achievement narrative’ is the ratio of the likelihood of clicking on achievement narrative against the likelihood of clicking the monetary narrative. Model 1 presents a baseline model that controls for narrative attributes only. Model 2 adds a control for audience attributes. Model 3 assesses the effect of monetary vs. non-monetary narratives. In Model 4, the non-monetary narratives are further divided into prosocial and achievement categories and compared to a baseline group comprising monetary narratives

### Prosocial narratives and achievement narratives perform equally well

Considering that non-monetary narratives outperformed monetary ones, we analyzed more closely the difference between prosocial and achievement motivations in climate change communications. We find substantively similar effect sizes for narratives describing the protagonists’ motivation that highlighted, for example, their concern for the environment (i.e., prosocial narrative) and for narratives that emphasized their sense of duty (i.e., achievement narratives). The likelihood of individuals clicking on narratives that describe prosocial motivations [OR = 1.51, *p* = 0.025, 95% CI = (1.05, 2.18)] is statistically indistinguishable from responses to narratives that explained the climate actions of a business owner protagonist with achievement motivations [OR = 1.56, *p* = 0.016, 95% CI = (1.09, 2.24)].

### Effects are generalizable across audiences

We investigated effect sizes for several audience characteristics to discern whether the observed effects were generalizable within our sample. When analyzing the effect of the perceived protagonists’ age (i.e., the estimated age of business owners featured in narratives) on audience responses to the three narratives we found no influence (Table 1). At the same time, individuals in older audience cohorts engaged more with all three narrative types. Further analysis revealed that this effect is driven primarily by audience members in the 65+ cohort that were—in line with other studies (Yousef et al. 2021)—more likely to click, regardless of the narrative that they were presented. Additionally, we matched audiences’ and protagonists’ age (e.g., younger protagonists with younger audiences), but found no effects on engagement. We also tested for interaction effects between audience age and narrative type and found no significant effects. In other tests of generalizability, we found that overall rates of engagement were similar for English- and French-speaking audiences and that effect sizes were consistent across both (for a detailed analysis, see the results report in Open Science Framework repository).

### Effects are robust across modes of engagement

We performed similar analyses with narrative clicks instead of link clicks as the dependent variable (see Table 2). Results were generally similar to our main findings: non-monetary narratives performed significantly better than monetary ones (non-monetary narratives: incidence rate ratio (IRR) = 1.64, *p* < 0.001, 95% CI = (1.35, 2.01)). However, in this case, we did find that the achievement narrative performed better than the prosocial narrative (prosocial narrative: IRR = 1.20, *p* = 0.113, 95% CI = (0.96, 1.51); achievement narrative: IRR = 1.96, *p* < 0.001, CI = (1.60, 2.41)).

**Table 2:** Generalized linear mixed-effects model predicting audience engagement as measured by narrative clicks (n = 5089)

Predictors	Model 5		Model 6		Model 7		Model 8	
	IRR (SE)	95% CI	IRR (SE)	95% CI	IRR (SE)	95% CI	IRR (SE)	95% CI
(Intercept)	0.15*** (0.07)	0.06–0.37	0.08*** (0.003)	0.03–0.19	0.06*** (0.02)	0.03–0.13	0.06*** (0.03)	0.03 – 0.14
Protagonist age	0.93 (0.21)	0.60–1.43	0.093 (0.19)	0.62–1.40	0.90 (0.18)	0.61–1.33	0.89 (0.28)	0.60–1.31
Protagonist gender	0.97 (0.27)	0.57–1.66	0.97 (0.25)	0.58–1.60	1.03 (0.25)	0.64–1.68	1.03 (0.25)	0.62–1.66
Language	0.99 (0.07)	0.86–1.15	0.98 (0.07)	0.85–1.14	0.97 (0.07)	0.84–1.12	0.97 (0.07)	0.84–1.12
Impression	1.02*** (0.00)	1.01–1.02	1.01*** (0.00)	1.01–1.02	1.01*** (0.00)	1.01–1.02	1.01*** (0.00)	1.01–1.02
Audience age			1.23*** (0.03)	1.17–1.30	1.23*** (0.03)	1.17–1.29	1.22*** (0.03)	1.17–1.29
Non-monetary narratives					1.64*** (0.17)	1.35–2.01		
Prosocial narrative							1.20 (0.14)	0.96–1.51
Achievement narrative							1.96*** (1.21)	1.60–2.41

*IRR* incidence rate ratio, *SE* standard error, *CI* confidence interval

*p < 0.05, **p < 0.01, ***p < 0.001. Model 5 presents a baseline model that controls for narrative attributes only. Model 6 adds a control for audience attributes. Model 7 assesses the effect of monetary vs. non-monetary narratives. Model 8 distinguishes between prosocial and achievement narratives compared to a baseline of monetary narratives

### Gender

We did not obtain clear-cut results when we analyzed the effect of the protagonists’ gender on how audiences responded. When analyzing link clicks, we found audiences responded about twice as much to achievement or prosocial narratives featuring female protagonists (non-monetary narratives: OR = 2.09, *p* = 0.003, 95% CI = (1.29, 3.40); prosocial narrative: OR = 1.94, *p* = 0.013, CI = (1.15, 3.28), achievement narrative: (OR = 2.23, *p* = 0.002, CI = (1.34, 3.71)). We found the opposite effect when analyzing narrative clicks: achievement and prosocial narratives that featured male protagonists performed significantly better (non-monetary narratives: IRR = 3.15, *p* < 0.001, 95% CI = (2.31, 4.30); prosocial narratives: IRR = 2.26, *p* < 0.001, CI = (1.61, 3.17); achievement narratives: IRR = 3.74, *p* < 0.001, 95% CI = (2.72, 5.13)).

## Discussion

In this study, we analyzed the capacity of narratives to create interest from businesses in climate actions. We conducted an online, real-world, large-*n* experiment to compare the effectiveness of different motivations in engaging business audiences in climate change discourse. Our research design encoded distinct monetary, prosocial and achievement motivations in three different narratives through close collaboration with SME owners taking climate action in business settings. We then showed these narratives to the Canadian SME target population in a naturalistic social media setting, and collected data on engagement.

Our research expands on past studies that have examined motivations for sustainable business practices from a purely economic perspective. These studies have shown how creating a business case for sustainability leads to (i) competitive advantage by increasing customer loyalty and willingness to pay, (ii) realizing greater efficiency, lowering costs or reducing risks in operations and supply chains, (iii) or spurring innovation and new product and service development (Reinhardt 1999; Vishwanathan et al. 2020; Yadav and Mankavil Kovil Veettil 2022). This perspective has been criticized for its instrumental logic which could alienate people pursuing sustainability because of genuine beliefs in environmental protection (Kaplan 2020; Rode et al. 2021). Indeed, laboratory experiments have highlighted how moral issues flavor executives’ advocacy for the business case for sustainability and why ideological beliefs have greater weight than evidence-based decisions among supporters (Hafenbradl and Waeger 2017). These results have generally formed two opposing viewpoints on whether opportunities for economic gains (Ditlev-Simonsen 2022) or non-monetary convictions (Strategic Direction 2020) are the core motivation driving businesses to engage with sustainability.

We found, contrary to what is typically assumed, that suasion through economic discourse is less effective even for business organizations. Employing prosocial and achievement motivations when communicating about climate actions is, based on our experiments, 55% more effective in generating interest than narratives that employ monetary motivation. Importantly, prosocial and achievement narratives both perform better than narratives expressing monetary motivations. These findings are robust across a variety of protagonist and audience characteristics, including age and language.

Our study design advances research on the effectiveness of monetary versus non-monetary motivations by overcoming constrains undermining past studies. Related research has been predominately restricted to laboratory settings (e.g., Hafenbradl and Waeger 2017; Hurst and Stern 2020), relied on observational studies to examine ongoing initiatives in real-world situations (Williams et al. 2017) or a combination of both (Forster et al. 2021). The experimental design we employed in this study overcomes these limitations by directly measuring responses to motivation encoded in storytelling. Moreover, it allowed us to control for audience and narrative characteristics in a real-world setting that remained uncontrolled in past studies. The three distinct, non-overlapping narratives that we tested in our experimental design demonstrate a feasible approach to disentangle the motivations that often convolute real-world stories of protagonists.

A key contribution of our research to the literature on sustainability communication is in shedding light on the effectiveness of distinct narratives in real-world settings. While personal motivations play a crucial role in people’s sustainability commitment (Schaefer et al. 2020), it remains challenging to differentiate between why people pursue actions initially, or what gets them interested at first, and the motivations that generate lasting commitment (Williams et al. 2017; Sloot et al. 2019). Analysis of motivations is often complicated by bias induced through retrospective reasoning and the fact that “people are not always aware of, or acknowledge, what motivates them” (Sloot et al. 2019, p. 9). The present research overcomes the drawbacks of self-report bias, by directly observing what kind of narrative is most effective in creating initial engagement with businesses on sustainability issues. We show that interest in stories about SME climate action is most strongly sparked through narratives outside of the widely used framing that suggest such initiatives as financially profitable or extending organizational longevity.

Narratives are most likely to strike a responsive chord, if they are woven into tangible stories and align with the lived experiences of target audiences (Hulme 2009; Williams and Schaefer 2013; Nisbet et al. 2015; Kahan and Corbin 2016; Wry and York 2017). For this reason, our research employed a geographically tailored approach to ensure close alignment between the developed narratives and audience characteristics. Therefore, the experiment in our study was limited to business audiences in Canada to align with the perspectives shared by the portrayed protagonists. This may limit the generalizability of our findings. To evaluate whether our results are generalizable to other countries and cultural settings, future research could apply the experimental approach we pioneered in other geographical areas.

Ultimately, this research reveals the most effective approach for sustainability communicators when approaching business audiences in the context of climate change. Importantly, our research focused exclusively on revealing what motivations are most effective in garnering initial interest in sustainability topics. Our research design did not assess where such communication leads to long-term commitment and implementation of climate actions. It would be valuable for future research to understand whether motivations that lead to initial engagement are also meaningful for generating lasting interest (Rode et al. 2021). In particular, the frequency of narrative communication and whether other kinds of engagements are needed to support audiences in committing to sustainability action are important areas for future research.

### Implications

In terms of guidance for sustainability communications, our study yields three practical implications. First, climate change communication directed towards businesses should not be limited to emphasizing potential monetary benefits. To be clear, our findings do not suggest that monetary motivations or mention of monetary issues in communications about climate action should be discontinued. Such communication will be effective for some audiences, and for many others, are likely to be complementary to other rationales for pursuing climate action (Asensio and Delmas 2015; Sloot et al. 2019; Forster et al. 2021). But they are not the most compelling and certainly not the only messages that should be employed. Our findings thus contribute to the reassessment that is underway regarding the assumption that people, especially those affiliated with businesses, are specimens of *homo economicus,* driven only by financial considerations (Wegner and Pascual 2011; Raworth 2017; Westman et al. 2019; Frank 2020).

Second, our results provide support for narrative-based communication approaches to promote desirable actions, even for business audiences. In particular, climate change narratives could become more effective by explicitly foregrounding prosocial and achievement motivations to engage audiences with climate action and, more broadly, sustainability. By focusing on pluralistic framings, sustainability messaging can benefit from a multiplicity of stories, moving beyond a single dominant narrative to employ complementary rationales for pursuing climate action (Luederitz et al. 2017; Linnér and Wibeck 2021).

Third, our research demonstrates the usefulness of articulating the challenges and successes of real people in advancing sustainability (De Meyer et al. 2021). Past research has proposed fictional stories to inspire climate actions (Sabherwal and Shreedhar 2022; Smalley et al. 2022). We advance this line of inquiry by demonstrating the practicality and effectiveness of employing real-world protagonists to communicate about climate actions. If narrative communication is employed in this way, it can benefit from authentic, factual human stories to create target audience-specific messages around real events and actions.

## Conclusion

In business settings, people are often assumed to act rationally with the sole aim of maximizing profits, thereby neglecting the multi-dimensional motivations at play when sustainability actions are undertaken. By testing different motivations encoded in non-overlapping narratives, we are able to show their relative leverage. Our findings advance previous studies that have assumed one-dimensional identities of audiences, associating a given actor with a single narrative. Instead, our research demonstrates the need for pluralistic understandings when dealing with the aspirations and motivations of societal actors. Employing complementary rationales, such as monetary, prosocial and achievement motivations, when engaging audiences in conversations about climate action would significantly advance the effectiveness of sustainability efforts. Future research should move beyond assumed narrative–actor associations, and examine the situational circumstances under which protagonists and audiences gravitate toward certain narratives.

The findings of our study advance narrative research as we demonstrate the effective use of relatable, real-world protagonists for highlighting challenges, achievements and questions encountered when people attempt to realize climate actions. Often sustainability communication is centered around success stories to entice audiences to join concerted efforts against climate change. The reality is that attaining sustainability goals—as is the case for realizing any course of action—is a challenging undertaking with setbacks, triumphs and conflicts. Our research placed the unique experiences of people at the center of narrative development to create authentic human stories that highlight real events and actions. Future research can advance this approach by moving beyond fictional characters and success stories to develop effective actor-specific messaging. To advance our work, research is needed on the particularities under which story elements that focus on failure, success and struggle resonate with specific subsets of audiences.

Our research has shown that efforts to encourage businesses to address climate change are likely to be more effective if multi-pronged and—alongside monetary justifications—the employed narratives emphasize prosocial and achievement motives. Considering the tremendous challenges in the transition to a low-carbon economy, our research offers a promising entry point for communication aimed at engaging people in ambitious climate actions.

## Data Availability

The datasets generated and analyzed by the current study are available in the Open Science Framework repository: https://osf.io/suyq5/

## Footnotes

1 The website has since been updated to incorporate various social media elements such as profile creation, storylines, forums, groups, among others. The layout of the website that was part of this research can be retrieved here: https://pivot-neon.vercel.app/home.
